# Reversible visible/near-infrared light responsive thin films based on indium tin oxide nanocrystals and polymer

**DOI:** 10.1038/s41598-020-69110-y

**Published:** 2020-07-30

**Authors:** Jian Wu, Chenzhong Mu, Jinglei Yang

**Affiliations:** 1 Key Laboratory of Magnetic Materials and Devices, Ningbo Institute of Materials Technology and Engineering, Chinese Academy of Sciences, Ningbo, 315201 China; 2 State Key Laboratory of Special Functional Waterproof Materials, Beijing Oriental Yuhong Waterproof Technology Co., Ltd, Beijing, 100123 China; 3 Department of Mechanical and Aerospace Engineering, Hong Kong University of Science and Technology, Clear Water Bay, Kowloon, Hong Kong, 999077 China

**Keywords:** Sensors and biosensors, Gels and hydrogels

## Abstract

In this study, we design a novel thermo- and photo-responsive nanocomposite film prepared by depositing indium tin oxide nanocrystals via the coating of amphiphilic copolymer on polycaprolactone substrates (INCP). The INCP film shows reversible surface morphology change properties by changing temperature as well as turning ON/OFF NIR laser. Especially, as the temperature changes from 25 to 75 °C, the film could regulate light transmittance from 75 to 90% across the visible and near-infrared region (500–1,750 nm). In addition, the film also exhibits excellent recycle and thermal stability at different temperature. Our results reveal that reversible surface morphology change properties are caused by curvature adjustment of film, which is owing to the coupling effect between copolymer and PCL with different thermal expansion strains. Our results suggest a possible strategy for the preparation of smart responsive materials in the future, which provides a reference for the development of new energy-saving materials.

## Introduction

As the world's growing energy crisis, efficient utilization of solar energy as clean energy faces great challenges in the future^1–3^. The development of smart materials is efficiently controlling solar light and has important practical significance of energy due to improving the energy efficiency of the built environment and hence to the reduction of energy consumption^4–6^. In particular, much attention has been paid to the thermo-responsive materials due to readily achievable using heat energy and simply preparing methods^7,8^. Previous studies have shown that nearly 50% of solar energy falls in the near-infrared (NIR) spectral region^9^. Thus, the development of new materials systems responsive to NIR has important practical significance. Although transition metal complexes and smart polymer are used extensively as thermo-responsive to visible light materials, a very common drawback is their relatively low stability because of evaporation of the solvent during repeated heating–cooling cycles^10–12^. Recently, vanadium dioxide (VO_2_) as a candidate smart material can change from a metallic state allowing infrared light through at high temperature to a semi-conductive state allowing visible light at low temperature. However, the high cost and difficulty of synthesis for VO_2_ limit their practical applications^13^. In addition, only a few intrinsic thermochromic materials that respond to visible and near-infrared light near room temperature have been reported to date^11–13^. Therefore, it is still a major challenge to develop new strategies to regulate the sensitive response of materials to temperature in the visible-near infrared region.

Currently, thermo-responsive inorganic nanoparticles coating polymer have attracted attention because of their promising potential applications in optical sensing and saving energy fields^14,15^. To this end, the studies for temperature-responsive nanoparticles/polymer materials mainly concentrate on Au, Ag, Co_3_O_4_ or Fe_3_O_4_ nanocrystals with covered polymer, such as polymer based on Poly(N-isopropylacry lamide) (PNIPAM), oligo (ethylene glycol) and so on^16–22^. However, the optical transitions of the above materials mainly are located in the UV–Vis spectrum range, nanoparticles-polymer extended into the near-infrared (NIR) spectral region has not been accomplished to our knowledge. Recently, Indium tin oxide nanocrystals (ITO-NC) have been reported to exhibit strong absorption peak in the Vis–NIR region. Especially, ITO-NC also show the strong absorption peak in the NIR region owing to their surface plasmon resonance (SPR) effect^23–26^. Although ITO-NC as an essential transparent conducting oxide have been applied in many fields, these ITO-NC coating polymer as smart materials remain a major chemical challenge^27–33^. On the other hand, the previous work have drawn attention to develop temperature responsive polymers in liquid condition. However, the solvent evaporation upon solar heating is a predictable problem that can shorten the cycle life of the irreversible systems or devices to limit their practice applications^34^. In the future, the application of temperature-responsive polymers in device field will also be restricted. Therefore, to develop innovative thermo-responsive materials in solid condition with stable property, while being a formidable challenge, is highly desirable. Previous studies have found that cross linked bilayer with Poly(N-isopropylacrylamide) (PNIPAM) containing polycaprolactone (PCL) exhibits reversible folding properties because of swelling and shrinking of PNIPAM.

Although some studies have reported PNIPAM and PCL as smart copolymers, the development of the inorganic nanoparticles coated with PNIPAM and PCL has not been reported^35–37^. Self-folding and self-wrinkling are also a universal phenomenon in nature. For example, the flower of oxalis rubra can open at daytime and self-folds at night. To study the mechanism of self-folding and self-curling phenomenon of plant is very meaningful to design novel smart responsive materials for application in saving energy fields.

Inspired by the aforementioned concepts, herein, we firstly prepared a novel thermo- and photo- responsive nanocomposite film by depositing tin oxide nanocrystals via the coating of amphiphilic copolymer (PMAO-PNIPAM) on polycaprolactone (PCL) substrates by dip-coated method. The film exhibits reversible optical regulation in visible and near-infrared region below 100 °C. We also investigate reversible surface morphology change process of the film by turning ON/OFF near-infrared light irradiation. Therefore, our study provides a new approach to build a solid-state photo responsive device for smart material in visible and near-infrared region.

## Results and discussion

**Figure 1:**
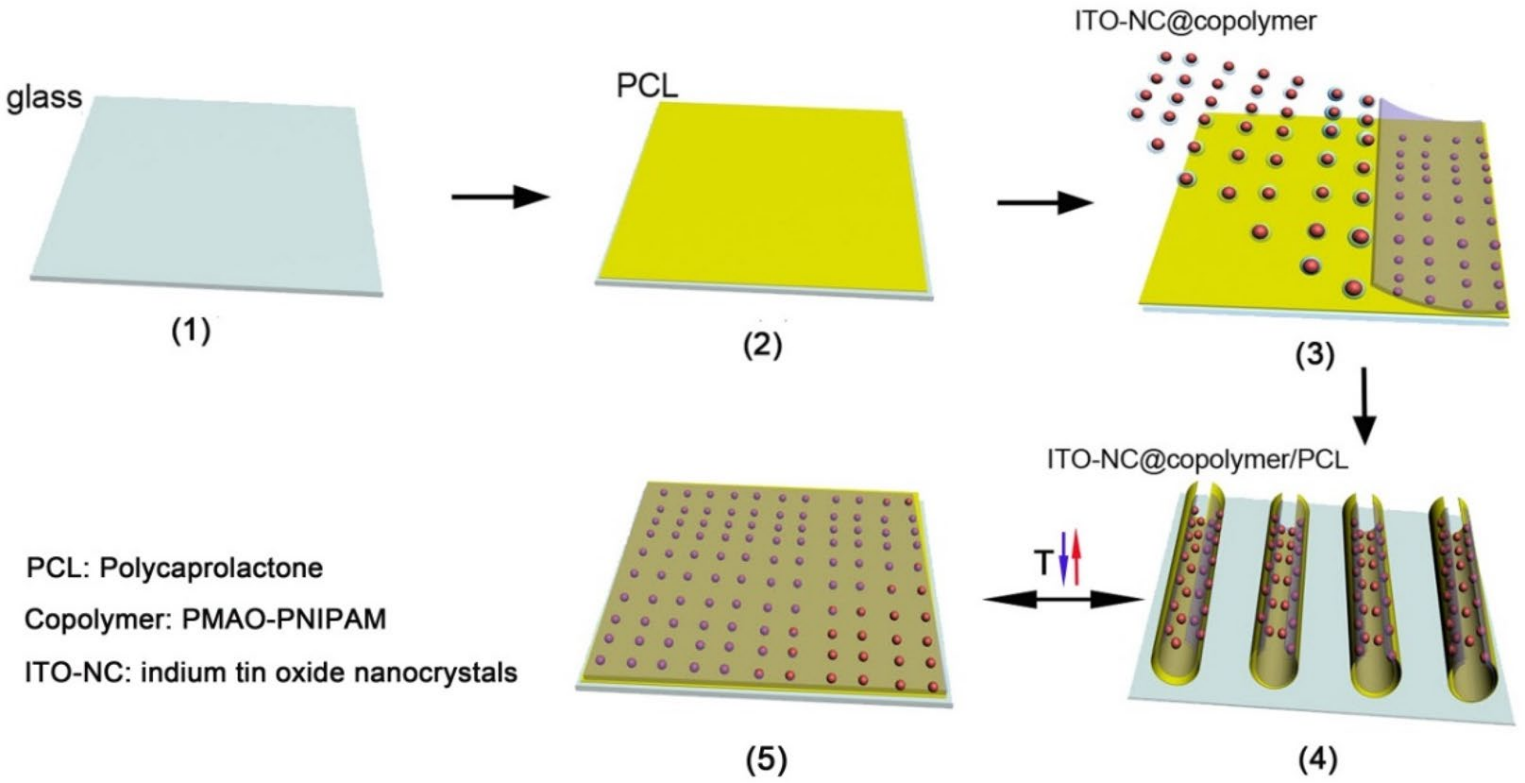
Schematic illustration of the procedure for demonstrating ITO-NC@copolymer/PCL (INCP) film, which exhibits thermo/photo-responsive properties changing with temperature.

**Figure 2:**
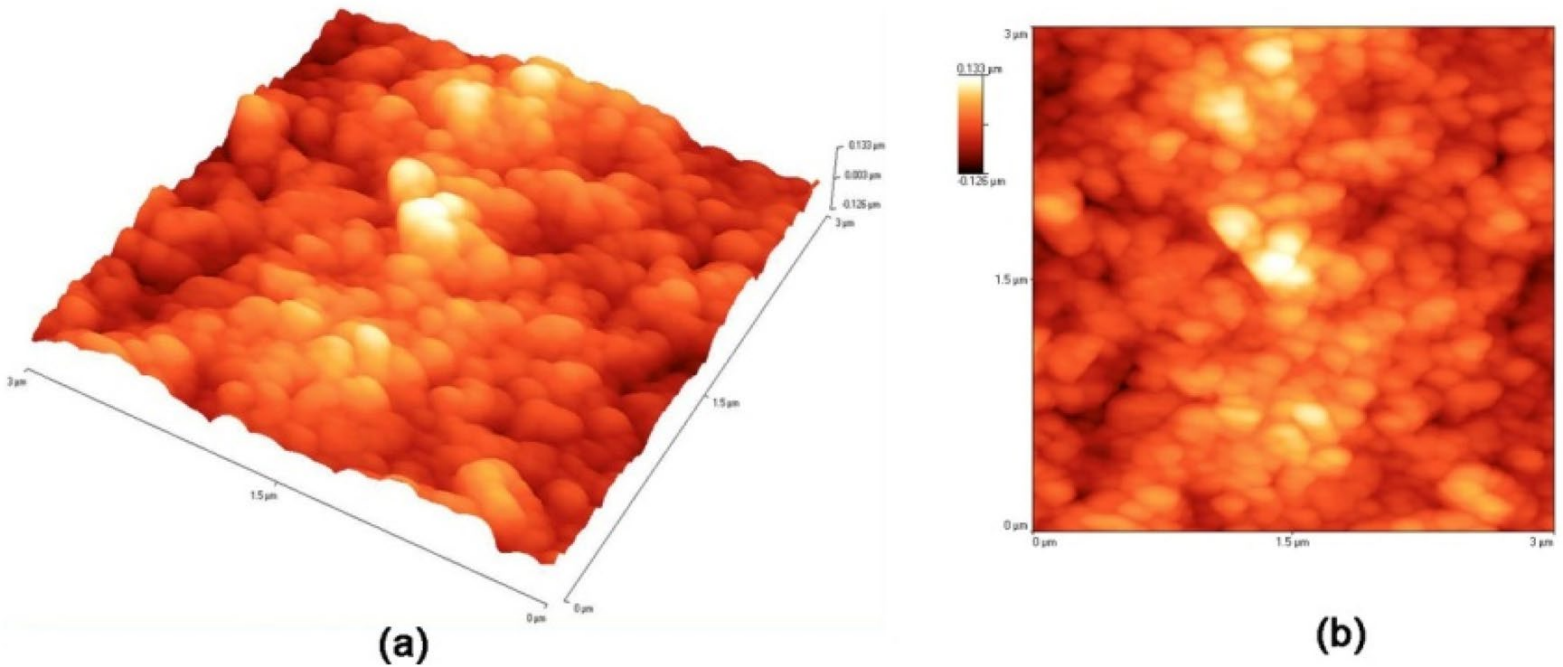
AFM surface image of INCP film.

Here we report the fabrication of thermo/photo-responsive ITO-NC@copolymer film. The film is synthesized by two-step method. In the first step, indium tin oxide nanocrystals (ITO-NC) and copolymer (PMAO-PNIPAM) can be synthesized following the previously reported method (see Experimental Section and Supplementary Information 1). The peaks of XRD patterns of ITO-NC can be indexed as the cubic In_2_O_3_, which is consistent with the values in the literature (JCPDS 06-0416) in Fig. S1c. TEM images of ITO-NC and ITO-NC@copolymer show that the nanocrystals are predominantly spherical in shape, which are nearly monodisperse and an average size of ~ 5–10 nm (Fig. S1a–b). ITO-NC exhibits strong absorption peak in the Vis–NIR region, as shown in Fig. S1d. Subsequently the surface modification of the ITO-NC@copolymer is carried out by ITO-NC coated with a layer of amphiphilic polymer PMAO-PNIPAM. The copolymer forms micelles in water, which encapsulate the ITO-NC. After coating with PMAO-PNIPAM, the ITO-NC modified with oleylamine are no longer soluble in cyclohexane but are highly dispersed in water. Next, the ITO-NC@copolymer is deposited from ethanol solution on the top of the polycaprolactone (PCL) film. The film can be called ITO-NC@copolymer/PCL (INCP) for short. Figure 1 illustrates the process and concept of the nanostructured of film. To investigate the surface morphology of INCP film, Atomic force microscopy (AFM) is carried as shown in Fig. 2. The AFM image shows that the surface thickness and roughness of INCP film are ~ 130–200 nm and 2.5 nm, respectively, which are applicable for photo-response and visible light transmittance (Fig. 2). Our results indicate that ITO nanocrystals and polymers are efficiently assembled into an INCP film, and the nanocrystals are uniformly dispersed in the polymer matrix, resulting in a nanoscale roughness of the film surface.

**Figure 3:**
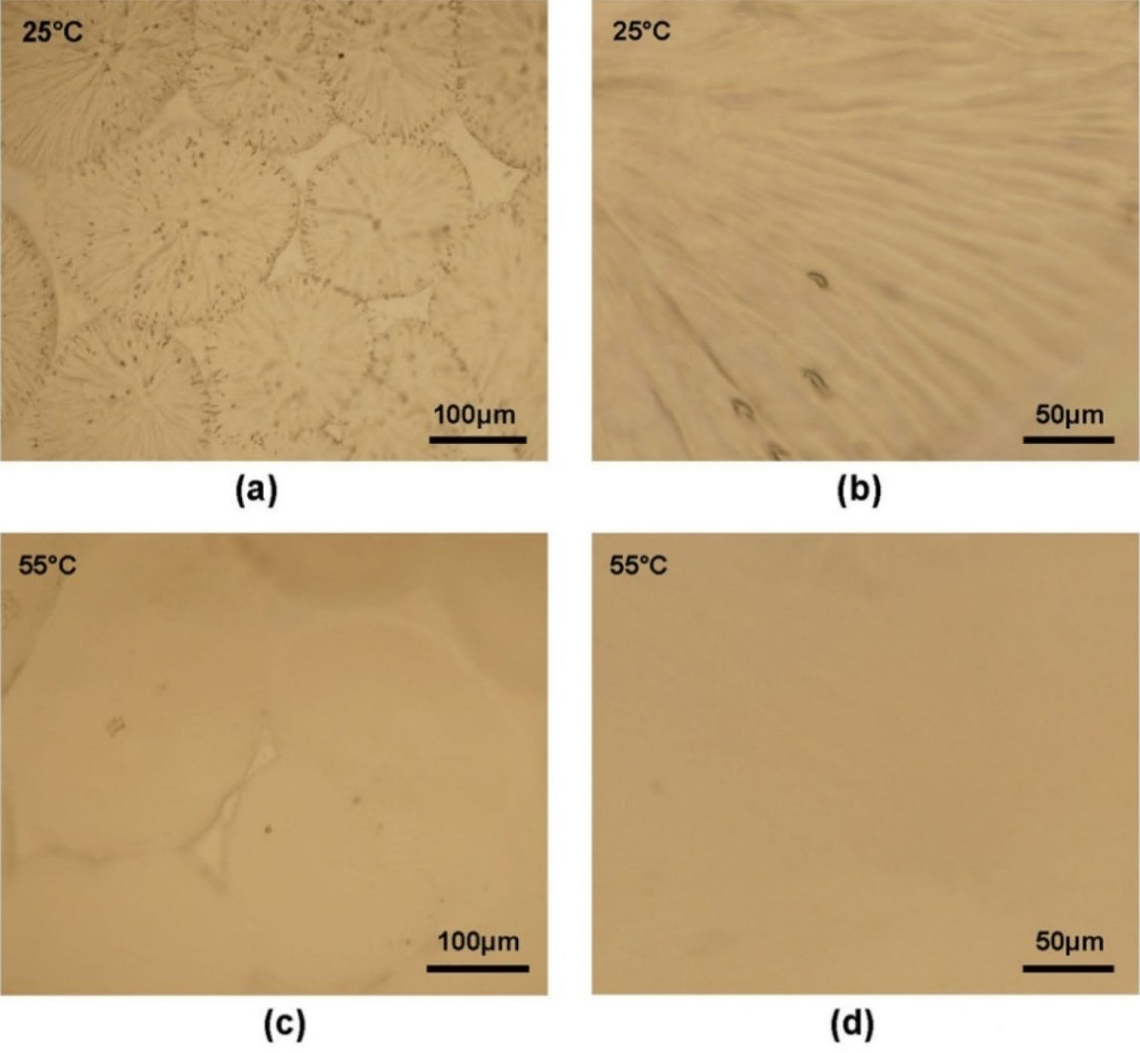
Bright field optical microscopy images of INCP film at 25 and 55 °C (**a**–**d**).

To check the macro-scale structure of the surface of the film, the surface morphology of INCP film are performed with optical microscopy images as shown in Fig. 3. Optical microscopy images reveal that the film has the shape of umbrellas including many tubes of ~ 5 μm diameters at room temperature, closely to those in previous reports of PNIPAM/PCL bilayers.^11^ Fig. 3 shows that the film is wrinkling at temperatures at 25 °C and becomes plane at 55 °C. Then, the INCP film can complete the reversible surface morphology change reversible changes of surface morphology process in 1 min. The bilayer film shows reversible surface morphology change properties in response to temperature which are probably contributed by the interaction between PCL and PNIPAM. The PNIPAM layer shows swelling and collapse with temperature change, while the PCL layer restricts swelling and collapse of PNIPAM layer. As a result, the umbrella-like INCP film show reversible surface morphology change with temperature change. Meanwhile, ITO-NC also expand/shrink with temperature change to tun transmittance of light. Thus, the above properties of INCP film are potentially useful as thermo-responsive smart material.

**Figure 4:**
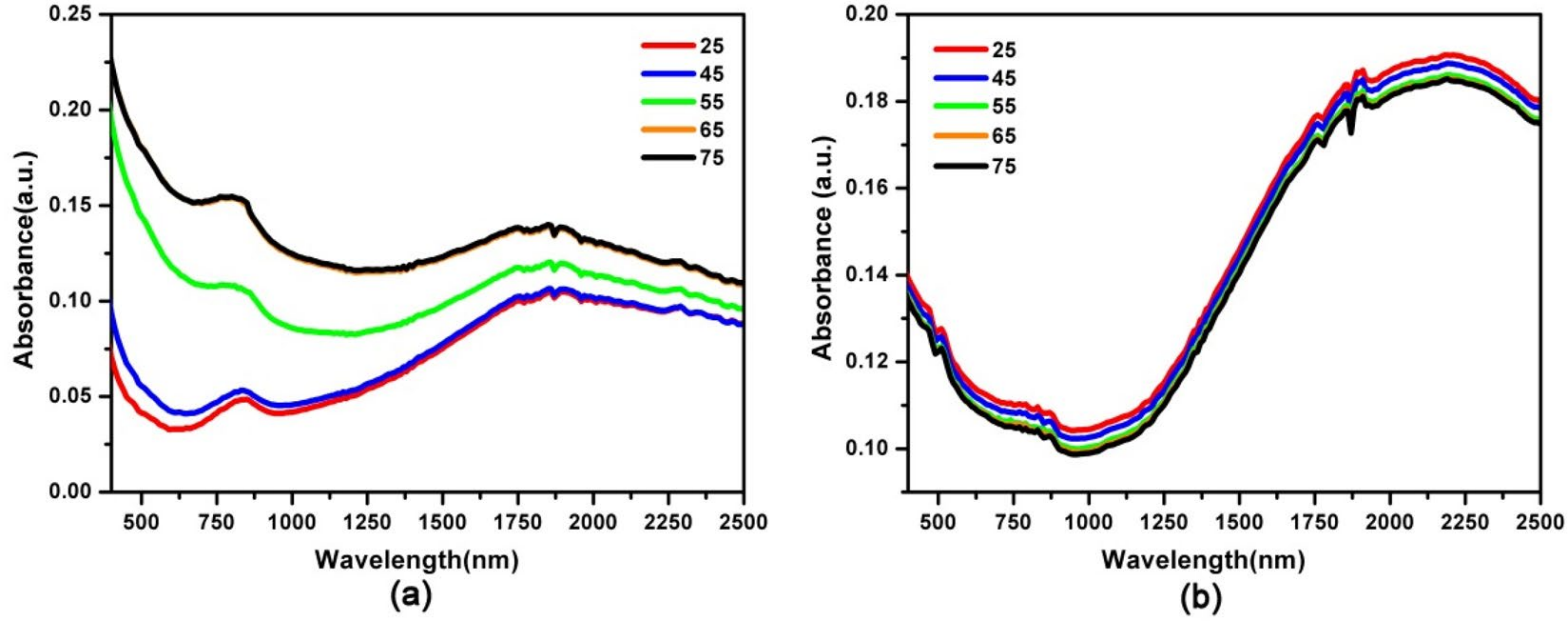
Absorption spectra of (**a**) ITO-NC@copolymer/PCL (INCP) films and (**b**) ITO-NC@ copolymer films at between 25–75 °C.

**Figure 5:**
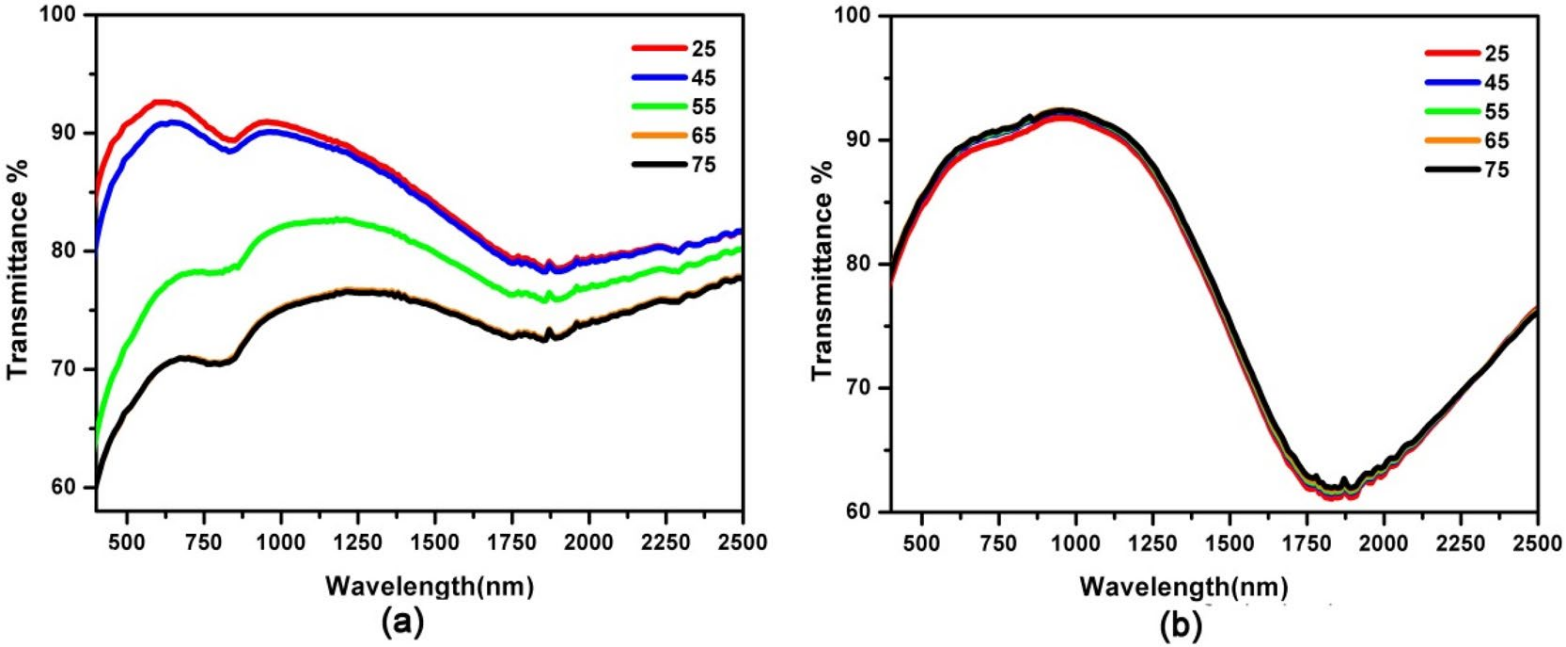
Transmission spectra of (**a**) ITO-NC@copolymer/PCL (INCP) and (**b**) ITO-NC@ copolymer films at between 25–75 °C.

To assess the optical properties of the film, samples are characterized by UV–vis-NIR spectrophotometer measurement. The film exhibits optical absorption in visible and near infrared region at room temperature with a broad absorption peak at ~ 1,200 nm. (Fig. 4a) The ITO-NC exhibit highly absorption efficiency in visible and near infrared region, which could be harnessed to boost the optical modulating ability in a broad wavelength range. Actually, similar absorbance features also exist in previously reported and are assigned to inter band transitions of indium tin oxide^26^. The absorption and transmission spectra of the film are investigated in the visible and near infrared region according to the temperature change (Figs. 4 and 5). The results show that the film exhibits thermo-responsive Vis–NIR absorption and transmission spectra during an increase of the temperature. The absorption intensities of the hybrid INCP film decrease with the increasing temperature from 25 to 75 °C (Fig. 4a). By contrast, the absorption intensity of INCP remains nearly unchanged when the temperature increases from 25 to 75 °C (Fig. 4b). Furthermore, the ITO film exhibits a NIR transmittance reversible change of 20% (from 95 to 75%) at 1,000 nm. However, the transmittance intensity of ITO-NC@copolymer nearly remains unchanged when the temperature increases from 25 to 75 °C. Therefore, the transmission properties of ITO-NC@copolymer are not temperature sensitive. Although PNIPAM on the surface of ITO-NC is thermal-responsive polymer, the conformational change of the copolymer without PCL is not significant enough to alter the optical absorption efficiency in the near infrared region.

**Figure 6:**
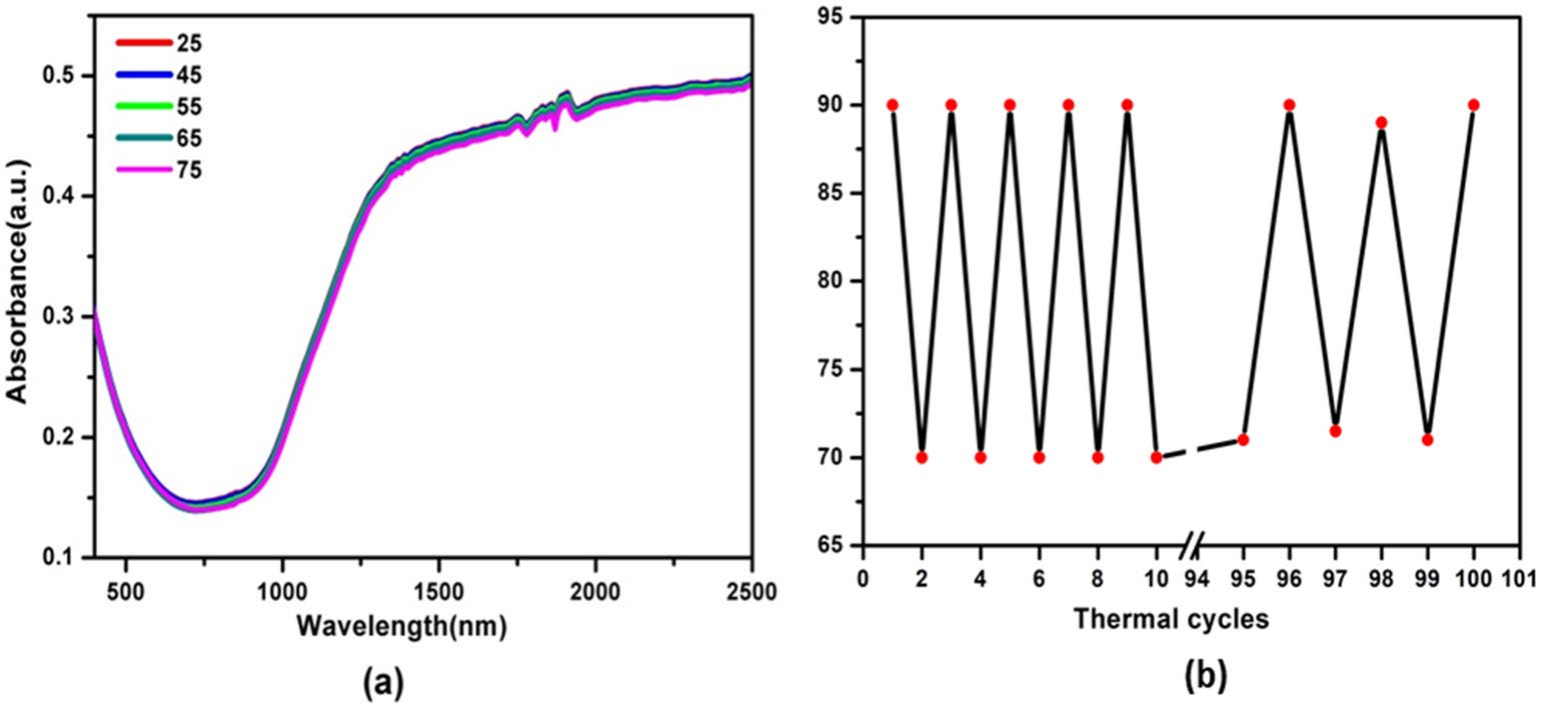
(**a**) Absorption spectra of ITO-NC@PEG/PCL (INPP) at between 25–75 °C. (**b**) Changes of transmittance of ITO-NC@copolymer/PCL (INCP) at λ = 1,000 nm during heating and cooling cycles between 25 and 75 °C.

In order to demonstrate the advantages of the ITO-NC@ copolymer/PCL film, it is tempting to evaluate the thermal-responsive performance of ITO-NC coated with polyethylene glycol (PEG), and then deposited on the PCL film (ITO-NC@PEG/PCL, INPP), as a comparison experiment. The INCP film is also found to exhibit qualitatively similar absorbance spectra with an intense NIR absorption peak. However, the absorbance spectra and intensity of INPP remain nearly unchanged before and after increasing temperature (Fig. 6a). Obviously, the transmission properties of the ITO-NC@PEG/PCL film are not temperature sensitive. Our results suggest that the conformational change of PMAO-PNIPAM is not significant enough to alter the film. In addition, the PEG has little effect on the temperature induced reversible surface morphology change of the film. Furthermore, to study the recycle stability of INCP film with temperature variation, we tested the light transmittance of INCP film at λ_max_ = 1,000 nm between 25 and 75 °C, respectively (Fig. 6b). The results demonstrate that film exhibits reversible durability after 100 heating–cooling cycles between 25 and 75 °C. The light transmittance of INCP film is also completely reversible between 70 and 90%.

**Figure 7:**
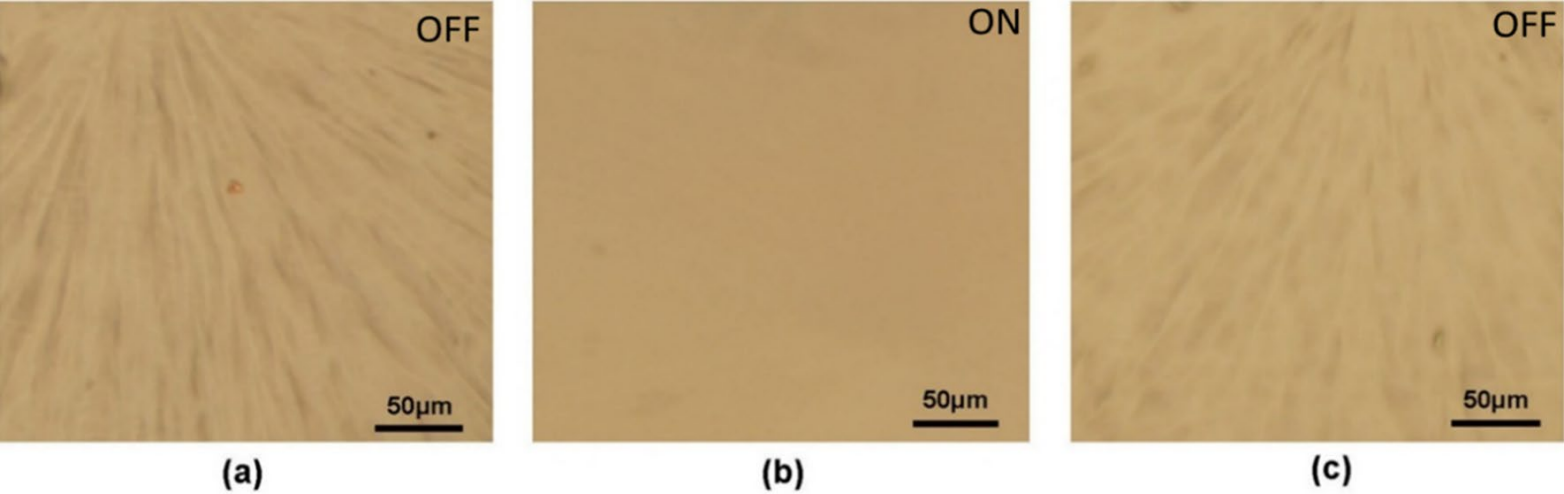
Infrared light-driven INCP film shown by optical microscopy images.

**Figure 8:**
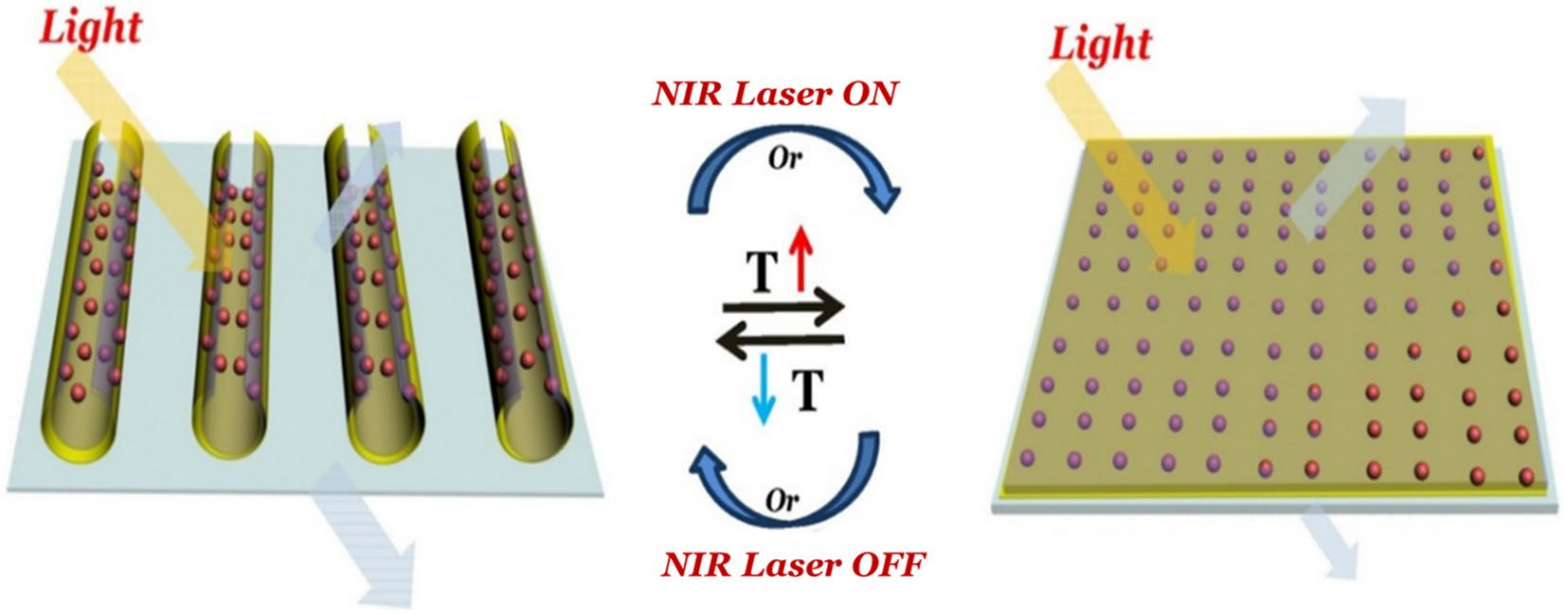
Schematic illustration of photo-responsive properties of INCP film induced by temperature or NIR laser.

In order to adjust the reversible change of surface topography more efficiently and conveniently, the films are not irradiated or irradiated with near infrared (NIR) laser (1,064 nm, 0.8 W/cm^2^) for 1 min. The optical microscopy images are used to directly observe the film before and after NIR laser irradiation. The film also exhibits reversible surface morphology change induced by infrared light, in similar with changing temperature, as shown in Fig. 7. The INCP film is in a wrinkled state at room temperature (Fig. 7a). Once the NIR laser is turned on, the film is completely spread on the glass substrate within 1 min (Fig. 7b). After removal of the NIR laser, the film recovers to its initial wrinkling state (Fig. 7c). The surface morphology change of fims is completely reversible for ON/OFF switching actuated by the NIR laser, in which the ITO-NC act as a nano-heater to raise the local temperature of the film via the light-heat conversion of ITO-NC. When NIR laser is applied, the result reveals that the temperature of ITO-NC increases by photothermal effect (Fig. 8). Compared to previously reported SWNT/PNIPAM, the film exhibits interested tube-shaped structure^38,39^. The newly provided nanocomposite film may open a door for the design and development of NIR-driven devices and machines, which would be useful in smart material.

## Conclusions

In conclusion, we have successfully designed a novel ITO-NC@copolymer/PCL (INCP) film, which exhibits reversible thermo/photo-responsive properties by changing temperature as well as taking infrared light irradiation. Particularly, the film could reversibly regulate light transmittance in the visible and near-infrared region with the increasing and decreasing temperature from 25 to 75 °C. In addition, the film also exhibits the reversible surface morphology change behavior controlled by NIR laser exposure or nonexposure. The findings demonstrate that coupling effect of ITO-NC@coplymer and PCL with different thermal expansion strains leads to the curvature adjustment of film. The thermo and photo induced nanocomposite film, with their visible and near infrared sensitivity, which provides a reference for the design of smart responsive materials in the future.

## Experimental section

### Reagent and chemicals

Chemicals. Indium (III) acetylacetonate (In(acac)_3_, 98%), tin (IV) chloride pentahydrate (SnCl_4_ 5H_2_O, 98%) oleylamine (70%), tri-n-octylphosphine oxide (TOPO, 90%), N-isopropylacrylamide (NIPAM), polycaprolactone (Mn 70,000–90,000), N, N-diisopropylethylamine, polyethylene glycol (PEG), polycaprolactone (PCL) and acryloyl chloride are purchased from Sigma-Aldrich. All chemicals are used as received without further purification.

### Preparation of samples

Fabrication of ITO-NC@copolymer/PCL film. Indium tin oxide nanocrystals (ITO-NC) and PMAO-PNIPAM, which comprise poly(maleic anhydride-alt-1-octadecene) (PMAO) and poly(N-isopropylacrylamide), are synthesized based on a previously reported procedure (see supplementary information for detailed experimental procedures). ITO-NC encapsulated in PMAO-PNIPAM microgels is prepared via a simple noncovalent functionalization route. In a typical process, the PMAO-PNIPAM solution (0.5 mL) is added to ITO-NC in chloroform (1.5 mL, 1.2 µmol/L) and magnetically stirred for 40 min. Upon completion, sodium borate buffer (pH = 11, 2 mL) is added to the mixture and subjected to vigorous agitation for one hour. The ITO-NC@copolymer in water solution is thereafter obtained after evaporation of chloroform. The buffer is exchanged to phosphate buffer (pH = 7.4) after 3 rounds of ultrafiltration at 3,000 rpm. Furthermore, the PCL film is deposited from polycaprolactone (PCL) in 5% toluene solution on a 1 cm^2^ glass substrate by dip coating. The solution of ITO-NC@copolymer in water is dip-coated on the PCL glass slide. The substrate is finally rinsed with deionized water to remove the excess polymer and dried with N_2_.

### Characterization

The optical properties of the film at different temperatures were measured by the Varian Cary 500 spectrophotometer with the temperature controller. The data are collected from 25 to 90 °C at a heating or cooling rate of 2 °C min^-1^. The transmission electron microscopy (TEM) images are obtained by a Hitachi Model H-800 instrument with a tungsten filament, using an accelerating voltage of 200 kV. High-resolution transmission electron microscopy (HRTEM) images are carried out on a JEOL-2010 transmission electron microscope at an acceleration voltage of 200 kV. The XRD patterns are recorded by using a Philips X’Pert Pro Super diffractometer with Cu Kα radiation (λ = 1.54178 Å). Atomic force microscope (AFM) images were carried out by Vecco di Innova. The temperature range investigated is from 17 to 70 °C. If not specifically mentioned in the text, an excitation wavelength of 430 nm is used. UV–Vis–NIR absorption and transmittance spectra are recorded by a Varian Cary 500 UV–Vis-NIR spectrophotometer along with a temperature controller.

## Supplementary information

Supplementary information.
