# Supplementary material for: Reversible visible/near-infrared light responsive thin films based on indium tin oxide nanocrystals and polymer
Source: Sci Rep. 2020 Jul 30;10:12808. doi: 10.1038/s41598-020-69110-y (PMC7393154; doi:10.1038/s41598-020-69110-y)
Supplement: Supplementary file 1 — Supplementary information. [file 41598_2020_69110_MOESM1_ESM.docx]

**Supporting Information**

**Reversible visible/near-infrared light responsive thin films based on indium tin oxide nanocrystals and polymer**

Jian Wu^1*^ Chenzhong Mu^2*^ and Jinglei Yang^3*^

^1^ Key Laboratory of Magnetic Materials and Devices, Ningbo Institute of Materials Technology and Engineering, Chinese Academy of Sciences, Ningbo, 315201, China.

^2^ State Key Laboratory of Special Functional Waterproof Materials, Beijing Oriental Yuhong Waterproof Technology Co., Ltd, Beijing, 100123, China.

^3^ Department of Mechanical and Aerospace Engineering, Hong Kong University of Science and Technology, Clear Water Bay, Kowloon, Hong Kong 999077, China.

*Corresponding author’s Email: [jwu@nimte.ac.cn](mailto:jwu@nimte.ac.cn); [muchenzhong@hotmail.com](mailto:muchenzhong@hotmail.com); maeyang@ust.hk

**S1. Experimental section.**

**1.1 Synthesis of indium tin oxide nanocrystals (ITO-NC).** In a 100 mL three-neck round-bottom ﬂask, 0.9 g of In(acac)_3_, 7.2 g of oleylamine and varying amounts of SnCl_4_·5H_2_O are mixed under the ﬂow of argon. The mixture is degassed and heated to 250 °C, and the reaction is allowed to proceed for 1 h under constant stirring in argon. The reaction mixture is subsequently cooled to room temperature. Two fractions of nanocrystals are obtained by the size selective precipitation. Large nanocrystals are ﬁrst precipitated by centrifugation at 3000 rpm for 10 min. Small nanocrystals are then precipitated from the supernatant by adding 20 mL of ethanol and centrifuging for 15 min. The precipitates of large nanocrystals are blue, while those of small nanocrystals are white. All nanocrystals precipitates are heated in melted trioctylphosphine oxide (TOPO), followed by precipitation with ethanol. This procedure is repeated three times to ensure the removal of surface-bound dopant ions. The obtained nanocrystals are easily dispersed in hexane.^1^

**1.2 Synthesis of PMAO-PNIPAM copolymer. (a) PNIPAM-NH_2_.** All reagents are used as received unless otherwise stated. The initiator 2, 2’-azobis(2-methylpropionitrile) (AIBN) is recrystallized twice from hot methanol. N-isopropylacrylamide (NIPAM) (24 mmol), 2-aminoethanethiol·HCl (0.24 mmol) and AIBN (0.024 mmol) were dissolved in anhydrous dimethylformamide (DMF, 10 mL). The solution was degassed by 3 freeze-pump-thaw circles. The glass reactor was sealed under vacuun. Polymerization was carried out at 75 °C for 22 hours. Upon completion, the product was precipitated in an excess of diethyl ether and washed with diethyl ether for 3 times. After drying overnight in the vaccum oven at room temperature,the crude PNIPAM-NH_2_ was dissolved in deionized water and subjected to dialysis (MWCO = 25 kDa) against copious amount of deionized water for one week with frequent water changes. PNIPAM-NH_2_ powder was thereafter collected by lyophilization. **(b) PMAO-PNIPAM**. PNIPAM-NH_2_·HCl (0.16 g) and triethylamine (1.6 mg) were dissolved in anhydrous chloroform (2 mL)and added to the chloroform solution of PMAO (1 mL, 0.8 mol/L) dropwise under agitation. The reaction was allowed to complete at room temperature for 12 hours. The PMAO-PNIPAM was collected under reduced pressure and the waxy solid was dissolved in chloroform (2 mL) to give a final solution with the monomer concentration of 0.4 mol/L.^2^


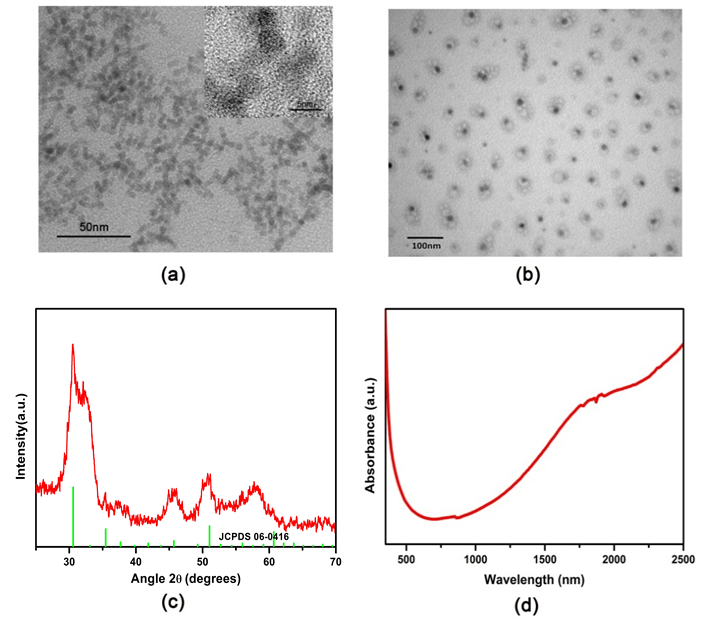


**Figure S1.** (a) Low magnification and high magnification TEM images of the ITO-NC. (b) TEM images of the ITO-NC@copolymer. (c) XRD pattern of ITO-NC. (d) Absorption spectra of ITO-NC.

**References**

1. M. Kanehara, H. Koike, T. Yoshinaga, T. Teranishi, *J. Am. Chem. Soc.* 2009, *131*, 17736-17737.
2. J. Qin, Y. S. Jo, M. Muhammed, *Angew. Chem. Int. Ed*. 2009, *48*, 7845-7849.
